# Quantification of LVEF≤35% misclassification by 2D-echocardiography as compared to cardiac magnetic resonance in coronary artery disease: implications for AICD therapy

**DOI:** 10.1186/1532-429X-14-S1-P212

**Published:** 2012-02-01

**Authors:** Diane J Parrington, Aramesh Saremi, Sudhakar V Girotra, Sandesh Dev, Charles Oh, Cynthia L Scott, Raymond Q Migrino

**Affiliations:** 1 Phoenix VA, Phoenix, AZ, USA

## Background

Cardiac magnetic resonance (CMR) is the noninvasive gold standard for evaluation of LV size/function due to its low interscan/intraobserver/interobserver variability. However, 2D echocardiography (Echo) remains the modality of choice to assess LV function due to ease of use, cost-effectiveness and ubiquity despite its known higher method variability. Randomized trials showed that automated implantable cardiac defibrillators (AICD) provide survival benefit in ischemic/nonischemic cardiomyopathy patients with LV ejection fraction (EF) ≤35%. It is therefore important to correctly classify patients by LVEF≤35% status to avoid improper placement or withholding of placement of AICD. We aim to quantify the misclassification of LVEF≤35% by Echo when compared to CMR in ischemic cardiomyopathy patients.

## Methods

Ischemic cardiomyopathy (LVEF<40%) patients had cardiac imaging at baseline and following 6 months of micronutrient supplementation on top of optimal medical therapy as part of a dietary supplementation study. Sixteen sets of same-day CMR and Echo scans were compared (from 8 subjects, all males, 71±8 years). LVEF was measured in CMR using cardiac-gated steady state free precession gradient echo cine and modified Simpson’s method, while biplane volumetric method was used in Echo. Correlation, ROC curve and kappa analyses were used.

## Results

LVEF was 30.7±9.4% (CMR) and 28.3±9.0% (Echo) (R=0.93, p<0.0001). CMR classified 10/16 while Echo classified 10/16 as having LVEF≤35%. Using CMR as gold standard, Echo misclassified 2 (12.5%) as having LVEF≤35% and misclassified 1 (6.2%) as having LVEF>35%. Kappa statistic for CMR and echo for LVEF≤35% is 0.59, denoting moderate classification agreement. By ROC analysis, an echo EF cutoff of 29% has 80% sensitivity, 100% specificity for detecting CMR LVEF≤35% with AUC of 0.92 (95%CI 0.67-0.99, p=0.001, see figure).

## Conclusions

Despite high correlation, Echo misclassified LVEF≤35% in 18.75% of cardiomyopathy cases versus CMR. With 1.2 million MI patients per year in the US and published data of 11% of MI patients having LVEF≤35% 90-days after MI, 132,000 patients potentially require AICD annually. Misclassification of LVEF≤35% by Echo has substantial implications for delivery or withholding of AICD therapy and the prognostic implications need to be formally studied. Short of using CMR in all post-MI patients, based on ROC analysis the misclassification can potentially be minimized by measuring CMR LVEF in patients with Echo EF≥30-40%.

## Funding

VISN 18 (Veterans Affairs) New Investigator Award.

**Figure 1:**
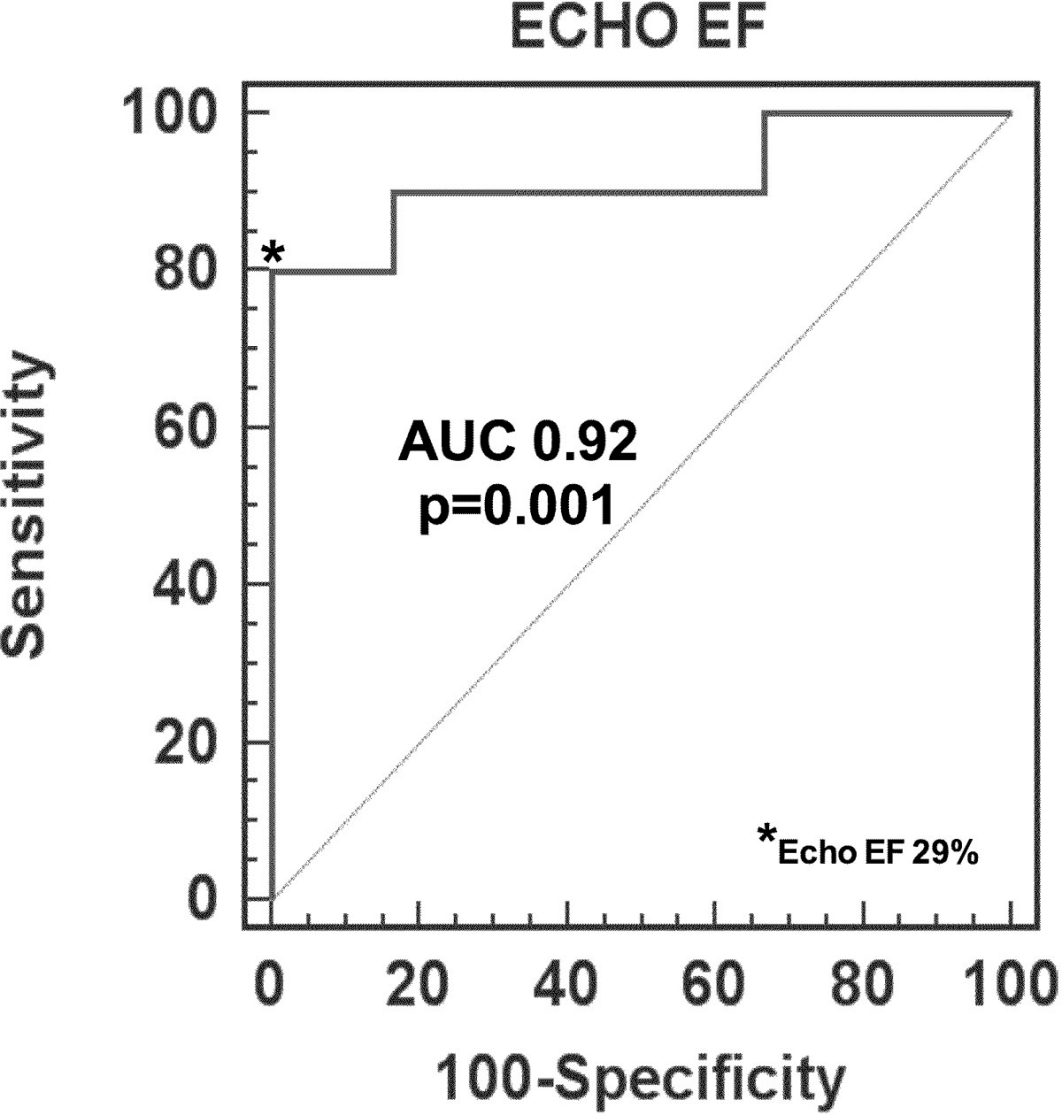
ROC curve analysis of Echo EF predicting CMR EF≤35%. Echo EF≤29% has 80% sensitivity and 100% specificity for predicting CMR EF≤35%.

